# Measuring Attentional Distraction in Children With ADHD Using Virtual Reality Technology With Eye-Tracking

**DOI:** 10.3389/frvir.2022.855895

**Published:** 2022-03-08

**Authors:** Jared D. Stokes, Albert Rizzo, Joy J. Geng, Julie B. Schweitzer

**Affiliations:** 1 MIND Institute, University of California, Davis, Sacramento, CA, United States; 2 Department of Psychiatry and Behavioral Sciences, University of California, Davis, Sacramento, CA, United States; 3 Center for Mind and Brain, University of California, Davis, Davis, CA, United States; 4 Institute for Creative Studies, University of Southern California, Los Angeles, CA, United States; 5 Department of Psychology, University of California, Davis, Davis, CA, United States

**Keywords:** ADHD (attention deficit and hyperactivity disorder), virtual reality, eye tracking, attention, distraction

## Abstract

**Objective:**

Distractions inordinately impair attention in children with Attention-Deficit Hyperactivity Disorder (ADHD) but examining this behavior under real-life conditions poses a challenge for researchers and clinicians. Virtual reality (VR) technologies may mitigate the limitations of traditional laboratory methods by providing a more ecologically relevant experience. The use of eye-tracking measures to assess attentional functioning in a VR context in ADHD is novel. In this proof of principle project, we evaluate the temporal dynamics of distraction *via* eye-tracking measures in a VR classroom setting with 20 children diagnosed with ADHD between 8 and 12 years of age.

**Method:**

We recorded continuous eye movements while participants performed math, Stroop, and continuous performance test (CPT) tasks with a series of “real-world” classroom distractors presented. We analyzed the impact of the distractors on rates of on-task performance and on-task, eye-gaze (i.e., looking at a classroom whiteboard) versus off-task eye-gaze (i.e., looking away from the whiteboard).

**Results:**

We found that while children did not always look at distractors themselves for long periods of time, the presence of a distractor disrupted on-task gaze at task-relevant whiteboard stimuli and lowered rates of task performance. This suggests that children with attention deficits may have a hard time returning to tasks once those tasks are interrupted, even if the distractor itself does not hold attention. Eye-tracking measures within the VR context can reveal rich information about attentional disruption.

**Conclusions:**

Leveraging virtual reality technology in combination with eye-tracking measures is well-suited to advance the understanding of mechanisms underlying attentional impairment in naturalistic settings. Assessment within these immersive and well-controlled simulated environments provides new options for increasing our understanding of distractibility and its potential impact on the development of interventions for children with ADHD.

## INTRODUCTION

Distractibility in daily life is now considered a major public health concern (*see* http://www.distraction.gov/) with distractions having a significant negative impact on attention. For children with attention-deficit/hyperactivity disorder (ADHD) distractibility is often extreme (Parsons et al., 2007; Friedman-Hill et al., 2010; Berger and Cassuto, 2014) and can lead to failures in academic achievement, task completion at home and school, challenges in interpersonal relationships (Paul, 2016), and likely higher mortality rates in automobile accidents (Chang et al., 2014) in adolescents and adults with ADHD. Distractibility by extraneous stimuli is so readily associated with ADHD, that it is one of the 18 items listed in the Diagnostic and Statistical Manual of Mental Disorders – 5 (DSM 5; American Psychiatric Association, 2013) ADHD criteria. Children with ADHD are likely to experience lifelong problems with distractibility and sustaining attention from childhood through adulthood (Paul, 2016). Distractibility can also be a significant contributor to the high cost associated with educating children with severe attentional impairments (Doshi et al., 2012). This cost can be seen in the need for children with ADHD to have academic accommodations, such as testing ADHD children in a room by themselves and/or extended test time, to mitigate the impact of distractibility on academic performance. Distractibility can also lead to other impairments in sustained attention (Adams et al., 2009; Slobodin et al., 2018) and executive functioning that likely have cascading consequences in the performance of daily activities. Given that ADHD is so common, with 5.9% of youth meeting diagnostic criteria for ADHD (Willcutt et al., 2012) and the lack of widespread accessible treatments with evidence for sustained, long-term effectiveness (Sonuga-Barke et al., 2014; Cortese et al., 2018), there is a need for better characterization of how distractibility impacts performance to better inform the development of targeted treatment approaches.

In order to more effectively identify the mechanisms that underlie distractibility, we need more objective and quantifiable measures of this impairment, beyond symptoms ratings, to better support research that aims to investigate cognitive and biological factors relevant to ADHD (Insel and Cuthbert, 2015). Currently, the standard of care for the assessment of ADHD involves interviews and standardized rating scales (Pliszka and AACAP Work Group on Quality Issues, 2007) and while these traditional measures are still critical, they are limited in their ability to assess and quantify distractibility with the sufficient objectivity needed to identify the underlying mechanisms associated with ADHD. This is exacerbated on a practical level by the difficulty experienced by a parent or teacher to quantify the degree of distractibility, particularly across different contexts. Consequently, challenges in quantifying distractibility likely limits our ability to systematically track change in response to treatment effects in clinical trials. The development of more objective measures associated with cognitive and biological functioning will ultimately enable us to identify targets for treatments of distractibility and is consistent with the Research Domain Criteria (RDoC) initiative (Insel and Cuthbert, 2015). Such efforts will serve to improve assessment (and ultimately intervention planning) across many diagnostic categories given that several mental and neurodevelopmental disorders are associated with impairments that are driven by distractibility.

A primary drawback of trying to assess distractibility and ADHD symptoms in the clinic or the laboratory setting pertains to the limitations that traditional methods have for quantifying problematic behaviors relevant to the activating stimulus conditions that may disrupt the performance of children with ADHD in everyday settings. Indeed, standard neuropsychological test results have been reported to have a relatively poor relationship with ADHD rating scale outcomes (Barkley, 2019). This may be in part due to the limited ecological relevance of traditional tests in the laboratory setting to actual performance in relevant everyday contexts (i.e., school settings). Comprehensive psychological and psychoeducational testing done properly would allow the examiner to see the distractibility of a student, which might be more reliable than the symptoms ratings by parents and teachers. Emerging evidence shows that virtual reality (VR) technologies can address the limitations of traditional methods by providing a more ecologically relevant experience that can be standardized across subjects (Bioulac et al., 2012; Lalonde et al., 2013). VR environments also enable strong experimental control, standardization of stimulus delivery, and response measurement. Recent research suggests that children’s task performance within a VR classroom environment generalizes better to real-world performance than traditional cognitive testing (Parsons et al., 2007; Lalonde et al., 2013; Díaz-Orueta et al., 2014). For instance, performance on a Stroop test delivered within a VR environment is more strongly associated with parent ratings of everyday executive functioning than performance derived from a traditional paper and pencil executive functioning measure (Lalonde et al., 2013). Similar research with a VR-delivered classroom environment suggested that a VR continuous performance task (CPT) was more effective at classifying children with respect to ADHD compared to a traditionally administered CPT (Adams et al., 2009) and this approach was more sensitive to medication effects (Díaz-Orueta et al., 2014). CPT tasks can be useful to understand differences between ADHD and non-ADHD children in performance as they also improve understanding related to sustained attention impairments, which may be related to underlying decreased in perceptual sensitivity and slower drift rates associated with ADHD. (Huang-Pollock et al., 2012). Children with ADHD also rate the VR cognitive testing and experience as more enjoyable than traditional tests (Pollak et al., 2009) and a more positive experience may result in children with ADHD persisting longer in a task. While the existing VR classroom research has thus far had an exclusive focus on the impact of external distractor on performance (as opposed to internal distractors), distractibility in classroom settings is commonly reported in children with ADHD. Thus, the use of VR to test performance in a context where distractors common to a real classroom environment, affords the opportunity for assessing the impact of distraction on cognition in a fashion that is not possible with traditional methods and may be more predictive of in class behavior.

The incorporation of eye-tracking within a VR headset is now becoming more practical and cost effective as many of these devices support eye-tracking capability as a method to improve visual resolution *via* a fovea rendering approach to the delivery of VR content (cf HP Omnicept).

However, until recently, the use of eye-tracking measures to better understand distractibility in ADHD has been relatively rare due to cost and complexity issues for implementation. Nonetheless, there is a history of research demonstrating eye-movement impairments in ADHD, partially reflective of a disrupted attentional system. For instance, researchers (Fried et al., 2014; Vakil et al., 2019; Lev et al., 2020), who used eye-tracking during CPT and Stroop assessments, demonstrated that persons diagnosed with ADHD produced more micro-saccades and fixated for longer durations on non-relevant regions, unrelated to target stimuli during task performance in comparison to typically developing (TD). The neural mechanisms of saccadic eye-movements are closely linked to that of attentional control (Moore and Fallah, 2001) and eye-movements have a long history of being used as a proxy for the locus of attention (Just and Carpenter, 1980; Levantini et al., 2020). For example, the probability of looking at a salient distractor, and the duration of the fixation, have both been used as metrics of distraction (Geng and DiQuattro, 2010; Born et al., 2011). More distractible individuals also tend to have a higher probability of looking at salient distractors and continuing to look at the distractor before disengaging attention (Fukuda and Vogel, 2010; Forster and Lavie, 2015). Eye-movements also indicate personal looking preferences (Haas et al., 2019), suggesting that idiosyncratic curiosity may contribute to task-irrelevant looking. Thus, looking behavior is highly sensitive to attentional priority, irrespective of whether the source of selection is task-based, physical salience, or personal interest (Forster and Lavie, 2008; Fukuda and Vogel, 2010).

While not employing eye-tracking measures, a recent paper by Mangalmurti et al. (2020) assessed how field-of-view (FOV) related to symptoms of ADHD within a virtual classroom during a CPT. The researchers measured changes in the FOV by recording head rotations to distractors in a VR classroom in children with ADHD to better understand what was driving attentional deficits. They found shifts in the FOV partly mediate the relationship between hyperactive/impulsive symptoms and impaired attentional performance, as well as information uptake. The relationship between FOV and hyperactive/impulsive as well as attentional performance highlights how the use of VR technology can inform our understanding of the broader phenotype of ADHD (i.e., inattention and hyperactivity/impulsivity). The authors point out that studies like theirs that assess the relationship between ADHD symptoms and cognitive impairments will be helpful in identifying remediation targets.

To investigate the impact of distraction on the performance of children with ADHD, we created a testing system that uses a VR headset to immerse a child within a virtual classroom. The first aim of this proof of principle study is to assess if our version of the virtual classroom with distractors could alter performance during periods associated with the presentation of distractors. Based on the assumption that real-life auditory and visual distractions will impair task performance, we hypothesize that the incorporation of environmental distractors within the VR classroom context will result in lower response attempts in all three behavioral tasks (i.e., Stroop, AX-CPT, Arithmetic/math). The second aim is to explore whether the incorporation of eye-tracking would provide a novel way to assess target engagement. Eye-tracking in the VR contexts potentially provides multiple measures to assess performance improvement that are novel in capturing attention and distractibility. Given that the incorporation of eye-tracking within a VR headset is still a relatively novel technique for cognitive assessment in this area, our analyses focus specifically on gaze duration on a virtual whiteboard (within the virtual classroom), comparing performance during periods of distraction versus no distraction. We hypothesize that the presence of environmental distractions in children with ADHD will decrease whiteboard eye gaze.

## MATERIAL AND METHODS

### Participants

A total of 20 children 8–12 (Mean = 10 years (SD = 1.65) years old participated in the study. The group was made up of two girls and 18 boys. Table 1 presents the demographic and clinical characteristics of the participants. The study was approved by the University of California, Davis Institutional Review Board (protocol number: 1170355), and we obtained verbal assent from each child and written informed consent from each participant’s parent. Recruitment was conducted through the Institute’s Subject Tracking System and advertisements using social media tools. Inclusion criteria included: Male or female age 8–13 years; Full Scale IQ equal or greater than 80; comfortable using a computer; elevated (T score ≥60) ratings of Cognitive Problems/Inattention or DSM Inattention scale scores on the Conners’ Parent or Teacher Rating Scale-3 (Conners, 2008) or Parent ADHD Rating Scale-V (ADHD-RS) (DuPaul and Power, 2016); Endorsement of four or more symptoms of inattention on the MINI-Kid (Sheehan et al., 2010). Note, because this study was focused on distractibility, we modified the DSM 5 criteria to accept children who might be considered to display subthreshold symptoms of ADHD to meet the diagnostic criteria, however, to be included the child was required to be rated as displaying significant degrees of distractibility by their parents. Exclusion criteria included: psychotic disorders (by parent report at phone screen), significant depression, autism (15 or > on Social Communication Questionnaire (SCQ)), visual or hearing impairment or any other disorder that may interfere with task performance; currently prescribed medication for ADHD or another psychiatric/behavioral diagnosis or starting behavioral/psychosocial treatment.

#### Diagnostic Procedure:

Parents/guardians were asked to complete a telephone screen before the first visit to determine initial eligibility. Two licensed psychologists with extensive experience diagnosing ADHD (JFD, JBS) evaluated initial phone screening data to determine eligibility for the study. Participants meeting the phone screen criteria were invited to proceed to the next phase of the study, which included an in-depth, in-person psychological evaluation. Measures to determine whether or not volunteers met inclusion/exclusion criteria included: Social Communication Questionnaire (SCQ) (Eaves et al., 2006), Children’s Depression Inventory (CDI-2) (March, 2013), Multidimensional Anxiety Scale For Children (MASC-2) (March, 2013), Columbia Suicide Scale (C-SSRS) (Posner et al., 2011), Conners 3 - Parent Rating Scale (Conners, 2008), Conners’ 3 Teacher Rating Scale (Conners, 2008), Clinical Global Impressions Scale (CGI), Wechsler Individual Achievement Test (WIAT: Numerical Operations and Word Reading - Wechsler, 2009), Weschler Abbreviated Scale of Intelligence (WASI-II; Vocabulary and Matrix Reasoning) (Wechsler, 2011), MINI-KID: Parent Version (Sheehan et al., 2010). A licensed psychologist (JFD) reviewed the diagnostic information to determine whether or not the inclusion and exclusion criteria were met based on the information gathered in the diagnostic process to determine final eligibility for the study.

All participants enrolled in the Virtual Reality Attention Management study registered at ClinicalTrials.gov (#NCT03221244, https://clinicaltrials.gov/ct2/show/NCT03221244). Participants and their families received $25 for the screening appointment and $50 for the baseline appointment, the focus of this paper.

### Apparatus and Experiment Design

The VR system was implemented using an AlienWare Aurora R7 gaming desktop computer: 8th Gen Intel Core i7-8700, 16GB DDR4 Memory, NVIDIA GeForce GTX 1080, Windows 10. Connected to the desktop computer was an HTC Vive head-mounted display (1080 × 1200 per eye pixel resolution, 110° field of view) with a SensoMotoric Instruments (SMI) manufactured integrated eye-tracking system. Eye tracking is based on the continuous measurement of the position of the eye with respect to the current visual field. In the HTC Vive, eye tracking uses a built-in infrared LEDs and a combination of corneal reflection and pupil localization to calculate the location of the eyes. This procedure is similar to standard desk-mounted video systems (e.g., by Tobii, SR Research). The calculation of which object the eye is pointed at is calculated with respect to the current field of view. In the VR context, information on the position of the eyes is combined with the depth of the virtual objects to construct a model of what was looked at in the virtual world. The HTC Vive eye-tracker sampled at 250 Hz and has a stated system accuracy of 0.5–1.1° of visual angle. Calibration was done with the built-in software using a 5-point calibration screen. Eye-tracking in this study is defined by the fixation location of the eyes. For example, fixation on the whiteboard is calculated as fixations within the boundary of the whiteboard object. Because the entire whiteboard is within the child’s field of view when looking forward (i.e., there are no objects that occlude the whiteboard) calculation of fixations on the whiteboard is straightforward. The VR Classroom was built using Unity 3D which included software that logged virtual object eye gaze data during the experiment. Headphones (Noot Products Stereo On-Ear Headset for Children) were used to present the auditory content.

Participants performed attention-demanding tasks while experiencing naturalistic auditory and visual distractors, relevant to those typically appearing in a real-world context, in a 3D simulated classroom. Our classroom was based on the original virtual classroom, first developed by Rizzo et al. (2000), which featured the simulation of a naturalistic environment, within which virtual test challenges and distraction stimuli could be systematically controlled to support performance testing across a range of highly controllable experimental conditions (Rizzo et al., 2004). Our classroom was designed to reflect more current classroom environments that included more racially and ethnically diverse child avatars within the environment.

**FIGURE 1 |:**
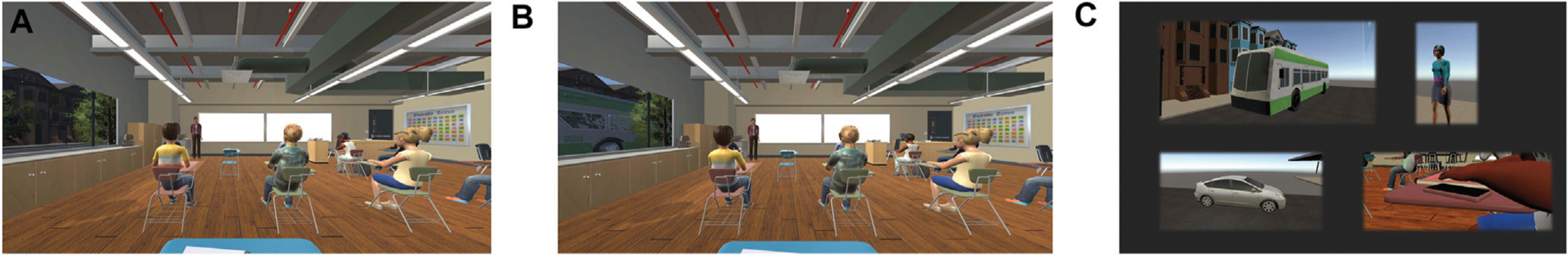
Virtual classroom and example distractors. **(A)** View of the classroom from the participant’s perspective with distractors off. **(B)** View of the classroom from the participant’s perspective looking out the window with the bus distractor on. **(C)** Sample of distractors - bus, car, and pedestrian walking by the window, and a phone ringing on another student’s desk.

Throughout each task block, the participant/wearer experienced distractions designed to be similar to real-life classroom distractors (*See* Table 2 for a full list of distractors) and similar to distractors used in prior VR classroom research (Rizzo et al., 2009) (*See* Figure 1). Generally, there were three types of distractors. *1*) Window: Distractors occurring outside the left window of the classroom. *2*) Student: Distractors attached to the student avatars. *3*) Phone: Two cell phones were positioned in the classroom. After the onset of the first block trial, the first distractor was triggered between 0 and 10s after the task started. Upon distractor completion, a 20 s cooldown period occurred. Immediately following, a distractor period was triggered, with the next distractor appearing within the following 0–10 s period. Each task block contained a randomly selected set of distractors. Most of the distractors featured both an auditory and visual component, and the duration of the distractor period was defined by the maximum duration across both components (*see* Table 2 for details on distractor types and durations).

### Task Descriptions

Participants were asked to complete three blocks each of a Stroop, Math, and AX-CPT in the virtual classroom while recording subject responses and eye movement data. Two participants did not complete a third block of the math task due to a technical error.

#### Stroop:

**FIGURE 2 |:**
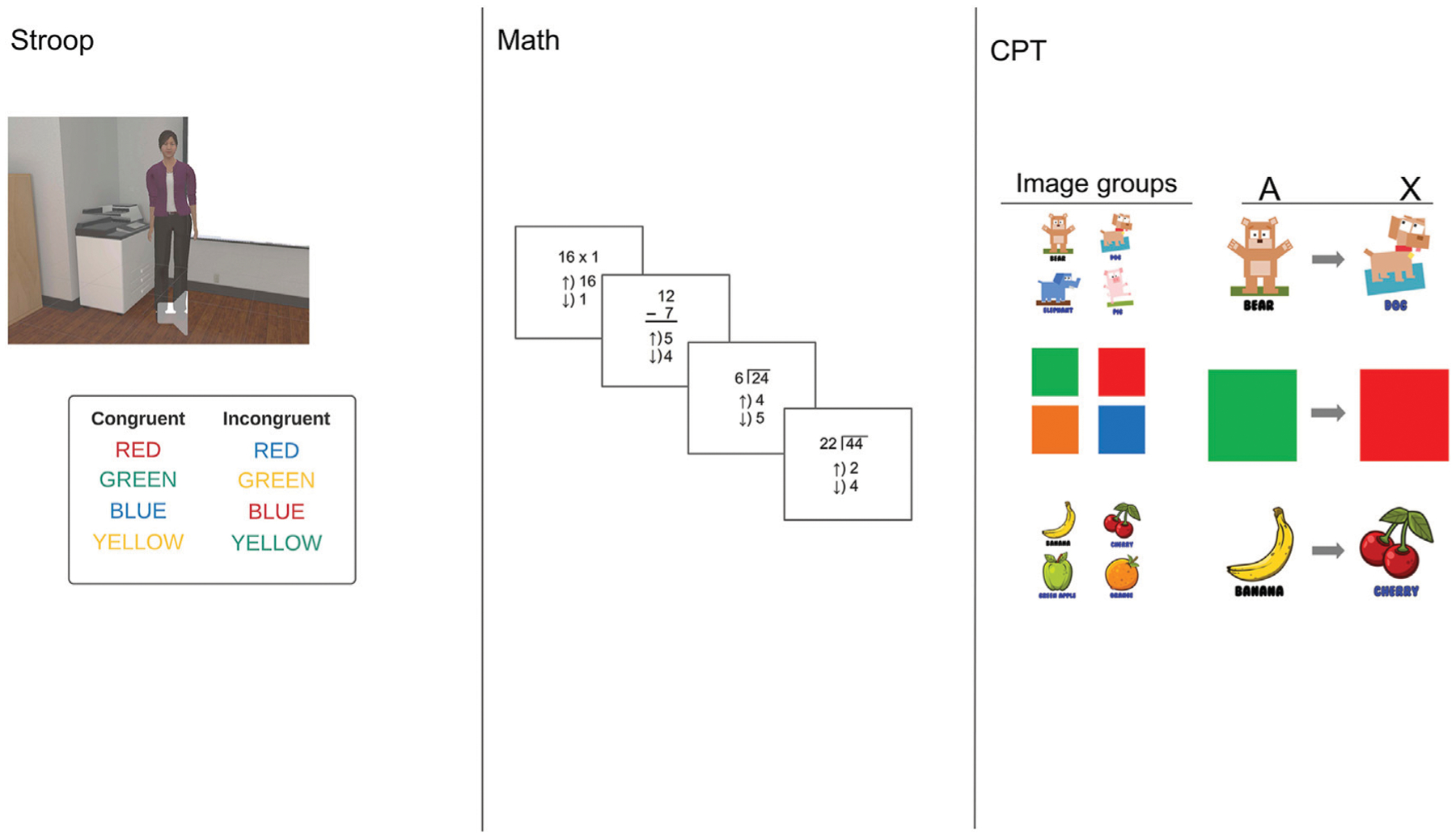
Illustrations of each task presented on the whiteboard at the front of the classroom next to the teacher (*see* leftmost panel). Participants were instructed to focus on the whiteboard tasks and complete as many problems as accurately as possible.

A trial-by-trial virtual Stroop test was presented on the whiteboard at the front of the classroom (Lalonde et al., 2013) (*see* Figure 2). The Stroop task is associated with executive functioning deficits in ADHD, and academic impairments, including greater risk for grade retention and lower academic achievement (Biederman et al., 2004). Stimuli were English language color words presented in the center of the whiteboard in red, blue, or green. Simultaneously, the teacher in the classroom verbally announced the color of stimulus ink. During congruent trials, the audio clip matched the color of the text ink. For incongruent trials, the audio clip color differed from the ink color. Participants were asked to identify whether the teacher was correct through a “Yes/No” response on the Vive controller. Pressing up on the Vive controller pad indicated “Yes” and a down press indicated “No.” Experimenters explained the instructions through a PowerPoint slide show prior to performing the tasks in the headset.

Each trial began with the presentation of a fixation cross in the middle of the virtual whiteboard (1000 ms). Word and auditory stimuli were presented after the fixation (2000 ms). Participants were required to respond within the 2000 ms stimulus presentation period. In each task block, participants performed 200 self-paced trials with a 50/50 ratio of congruent and incongruent trials.

#### Math problems:

The math task was an adaptation of a task with 7–9-year-olds (Rosenberg-Lee et al., 2011). Math problems were selected as a task to simulate a real-world task that elementary children encounter in real class settings. Math performance is considered highly dependent on attention and thus, it was hypothesized that it would be affected by distractibility. ADHD is associated with several measures of academic impairment, including in math performance (Loe and Feldman, 2007; Efron et al., 2014), however, another primary reason to include a math task in the VR context is that they are frequently used to assess treatment effects in ADHD clinical trials, particularly as a measure of completion, rather than a measure of skill (Wigal and Wigal, 2006) Indeed, math productivity and accuracy have also been reported to be sensitive to medication effects in ADHD (Faraone et al., 2007). We chose to include simple and complex addition trials, but not math problems that required sophisticated math problem-solving. Participants indicate their answer on a touchpad whether the equation is correct (e.g., “3 + 4 = 8”). Answers are correct in 50% of trials.

#### CPT:

Participants completed three blocks of the CPT, which was modeled after AX-CPT methodology used to assess sustained attention in ADHD populations in prior studies (Parsons et al., 2019). To make the task more accessible to children, we used images in place of the typical letters of the alphabet. Participants were instructed to view a series of images and to press the appropriate response button when viewing the “X” image (e.g., dog) if preceded by the “A” image (e.g., bear) (*See* Figure 2 for images used). Each block featured a separate group of images using developmentally appropriate stimuli such as animals, fruits, and colors.

### Task Metrics

#### Whiteboard fixation percentage.

To assess the extent that attention was directed to the task, we calculated the percentage of time that eye gaze (i.e., fixations) was on the whiteboard during a task period of interest. An invisible interest area was drawn around the whiteboard as if in a standard sized classroom (*see* Figure 1). Fixations within that boundary area are considered on-task. All other fixations were considered off-task. To assess our hypothesis that gaze on the whiteboard and task performance would both diminish during distractions, we extracted the percentage of fixation time on the whiteboard within three time-bins locked to the onset of the active distractors (−5000-to-0 ms pre-distractor onset, distractor onset-to-5000 ms post-distractor onset (bin 0), and 5001-to-10000 ms post-distractor onset (bin 1).

#### Response rate.

Response rate was used as a performance measure of overall attentiveness. Response rate, or the number of responses made in each period of interest was measured separately for each task. Response rate was chosen as the metric of attentiveness and was used rather than response accuracy because the children varied idiosyncratically in how difficult they found the math problems. Time periods of interest were defined by distractor-off and distractor-on periods. Distractor-on and distractor-off periods were identified and binned within each task block. Distractor-off periods were defined by the default classroom setting. Distractor-on periods were defined as those during which a distractor was active in the environment during that task period.

#### Gaze distraction index.

To illustrate the impact of distraction on eye gaze, a gaze distraction index was calculated by taking the difference between post-distractor bin whiteboard fixation percentage (1–5000 ms after the distractor onset) from the pre-distractor bin and dividing by the pre-distractor bin fixation percentage.

#### Performance distraction index.

To index the effect of the distractors on task performance, a performance distraction index was calculated by subtracting the mean task block response count from the distractor-off condition from the distractor-on condition and then dividing that difference by the mean response rates for both conditions. Doing this provides a value that indicates the change in the proportion of problems completed given the presence of a distractor compared to distractor-off periods. The distraction index is similar to omission errors and captures lapses in attention, a measure frequently elevated in ADHD (e.g., Buzy et al., 2009), but in the context of presenting known distractors.

### Data Analyses

Repeated-measures ANOVAs with post hoc *t*-test comparisons were used to analyze differences in whiteboard fixation for each time-bin, as well as differences in response rate in distractor-off and distractor-on task periods. Task differences in distraction index were investigated with repeated-measures ANOVAs. The relationship between gaze and performance distraction indices was analyzed with Pearson’s correlation and a linear mixed model. Pearson’s correlation was also implemented to analyze consistency within the gaze distraction indices. Statistical comparisons and correlations were conducted in Python using the Pingouin package (Vallat, 2018). Linear mixed modeling and significance testing were conducted in R using the lme4 (Bates et al., 2015) and lmerTest (Kuznetsova et al., 2017) packages, respectively.

## RESULTS

### Eye Gaze

**FIGURE 3 |:**
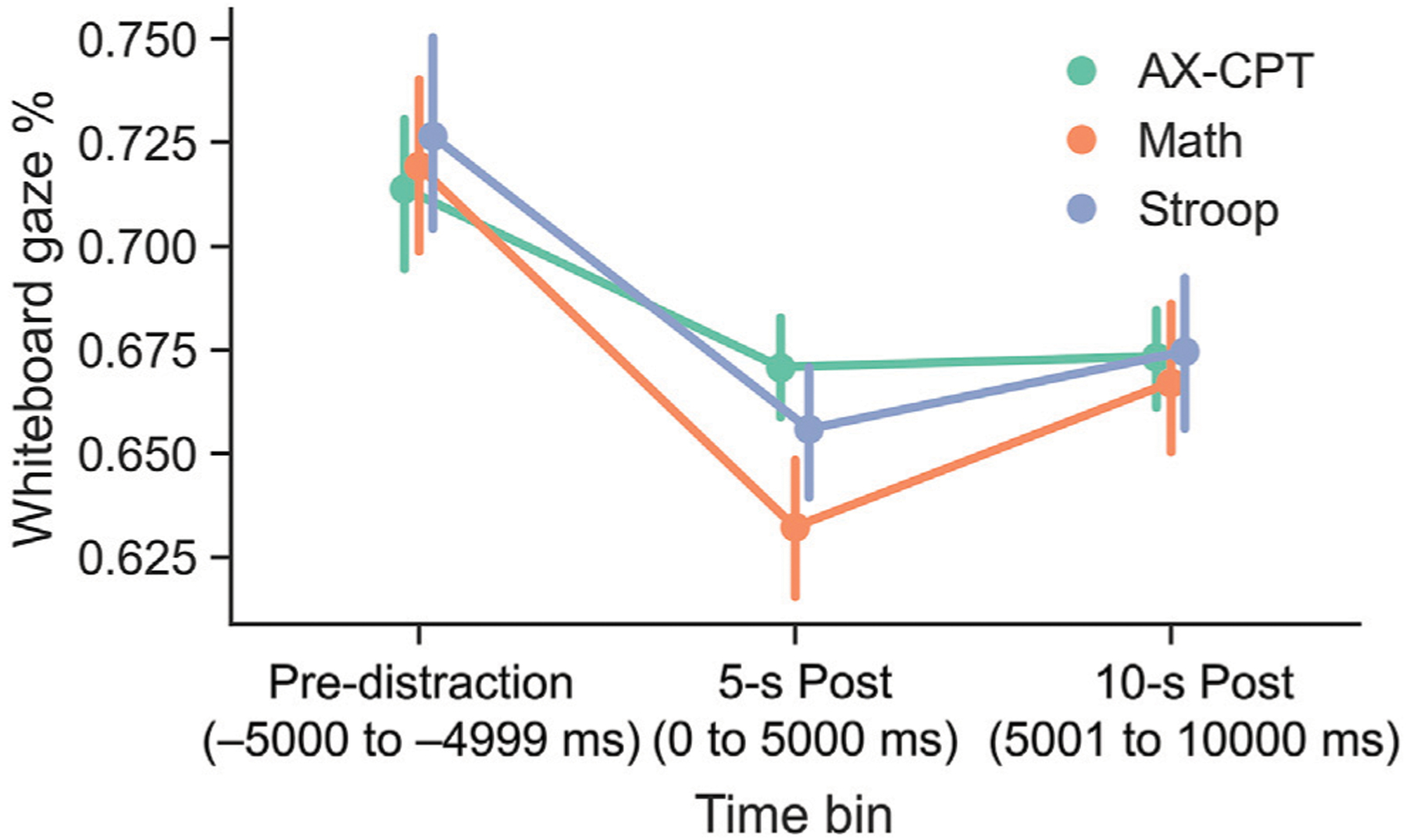
Average percentage of time spent looking at the whiteboard as a function of 5 s time bins relative to the onset of a distractor objects. The “pre-distraction” time bin includes the 5 s just prior to the distractor onset, “5-s post” includes the first 5 s after distractor onset, and the “10-s post” time bin includes the 5–10 s period after distractor onset. Data demonstrate that the percentage of time the eyes were fixated on the whiteboard was uniformly high during all three tasks before the onset of a distractor. Looking at the whiteboard decreased significantly after the distractor onset and this was sustained or up to 10 s after the distractor occurred. The pattern suggests that distractor events reduced looking at the whiteboard that was sustained over time, even when the distractor itself was no longer actively present (*see* text). Error bars represent 95% confidence intervals after removal of between-subject variability.

The primary hypothesis was that the onset of distractors would cause a reduction in the amount of time that participants looked at the whiteboard. To evaluate this hypothesis, we investigated whiteboard gaze percentage in three bins: Pre-distractor onset: −5000 ms to 0 ms before the distractor onset; post-distractor onset bin 0; 0–5000 ms after the distractor onset; post-distractor onset bin 1; 5001–10,000 ms after the distractor onset. Whiteboard gaze percentage was calculated by extracting the total dwell time on the whiteboard divided by the period duration. Figure 3 illustrates that the time spent looking at the whiteboard decreased significantly from the pre-distraction to the post-distraction periods. A one way repeated-measures ANOVA with time-bin modeled as a repeated measures factor and whiteboard gaze percentage as the dependent measure was conducted for the data in each of the three tasks. The results were significant in all three tasks (AX-CPT: *F*(2,38) = 6.78, *p* < 0.005, MS = 0.012, $\eta_{p}^{2}=0.26$; Math: *F*(2,38) = 12.86, *p* < 0.0005, MS = 0.038, $\eta_{p}^{2}=0.40$, Stroop: *F*(2,38) = 8.49, *p* < 0.005, MS = 0.027, $\eta_{p}^{2}=0.31$. (*See* Supplementary Table S1 for results in Table format.)

Subsequent *post hoc* pairwise t-tests confirmed the effect of time-bin was due to significantly greater looking at the whiteboard during the 5000 ms time-bin prior to the onset of the first distractor compared to the first post-distraction bin (AX-CPT: *t*(19) = 2.91, *p* < 0.05, Cohen’s d = 0.34; Math: *t*(19) = 4.66, *p* < 0.0005, d = 0.57; Stroop: *t*(19) = 3.90, *p* < 0.005, d = 0.48) and second (AX-CPT: *t*(19) = 2.66, *p* < 0.05, d = 0.33; Math: *t*(19) = 2.74, *p* < 0.05, d = 0.38; Stroop: *t*(19) = 2.53, *p* < 0.05, d = 0.34) 5000 ms time-bins following the distractor onset. There was no difference in looking behavior between the first and second post-distraction time-bins in two tasks (AX-CPT: *t*(19) = −0.3, d = −0.02; Stroop: *t*(19) = −1.33 *p* = 0.20), d = −0.12, but there was a significant increase in the math task (Math: *t*(19) = −2.56, *p* < 0.05, d = −0.21). These data suggest that the onset of the distractors was followed by a decrease in attention to the whiteboard for the next 10 s, although attention to the whiteboard may have recovered faster for the Math task compared to the other two. The fact that similar patterns were found in all three tasks indicates that the effect of distraction on looking behavior was independent of the task being performed.

### Task Performance

**FIGURE 4 |:**
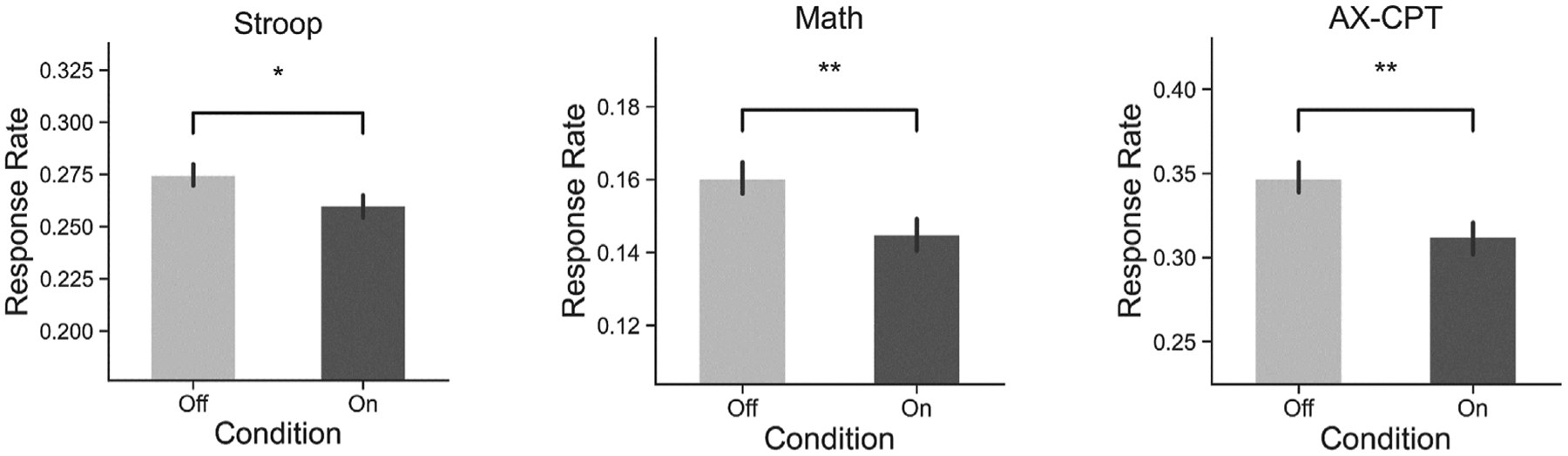
Bar graphs showing the proportion of task-based responses during distractor On or distractor Off periods of time. Error bars represent 95% confidence intervals after removal of between-subject variability. Statistical significance denoted by “*” for *p* ≤ 0.05 and “**” for *p* < 0.001. In all tasks, the number of problems responded to decreased when distractors were present compared to when they were absent. The consistency across tasks suggests that distractors interfered with performance irrespective of which task was being performed.

We further evaluated the effectiveness of the classroom distractors to impair on-task attention with response rate, that is, the number of responses made to problems during a given period of time. A repeated-measures ANOVA of response rate showed a main effect for distractor state (*F*(1,19) = 19.75, *p* < 0.0005, MS = 0.014, $\eta_{p}^{2}=0.51$). Post-hoc analysis showed that response rate was lower during distraction-on periods than distraction-off periods for all three tasks (Stroop: t(19) = 2.61, *p* < 0.05, d = 0.28; Math: t(19) = 3.32, *p* < 0.005, d = 0.31; AX-CPT: t(19)= 3.54, *p* < 0.005, d = 0.42) (Figure 4). These results suggest that the presence of virtual distractors resulted in lower overall responses in all three tasks.

### Eye Gaze × Task Performance

**FIGURE 5 |:**
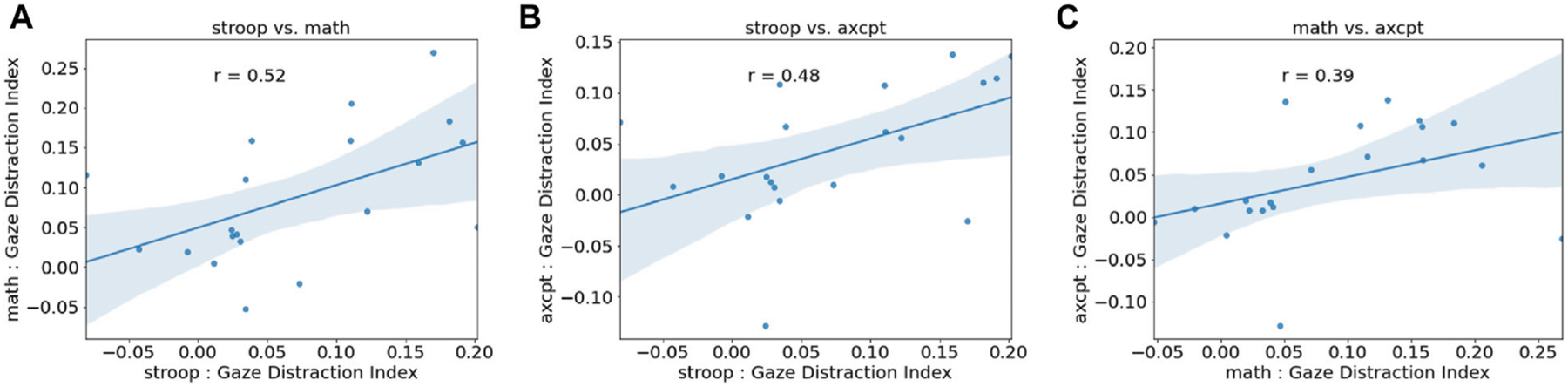
Distraction indices by task calculated as change in behavior once a distractor appeared. **(A)** Gaze distraction index, calculated as the change in percent time spent looking at the whiteboard once a distractor appeared, during each task. There were no differences in gaze distraction by task, ns = not significant. **(B)** Performance distraction index, calculated as the change in the percent of problems responded to once the distractor appeared, during each task. **(C)** Significant correlation between gaze and performance distraction indices indicates that participants who looked away from the whiteboard more when distractors appeared also completed fewer problems. This provides an empirical link between eye gaze on the whiteboard and ability to complete problems, across all task types.

To further investigate the effect of distraction on eye gaze, we calculated a gaze distraction index which represented the percentage decrease from pre-distractor time-bin (bin 0) to the first post-distractor time-bin (bin 1) (*(bin 0 gaze percentage – bin 1 gaze percentage)/bin 0 gaze percentage*) and was calculated separately for each distractor in all three task blocks and all tasks. To examine the consistency of the measure within participants, we looked at between task correlations of gaze distraction index which indicated significant/trending positive correlations between AX-CPT, math, and Stroop tasks (Figure 5). Additionally, differences in gaze distraction index between tasks were also examined using a repeated-measure ANOVA, which showed that the task was not significant (*F*(2,19) = 1.48, *p* = 0.24, MS = 0.017, $\eta_{p}^{2}=0.072$).

Next, we calculated a performance distraction index (*(distraction-off response rate – distraction-on response rate)/mean response rate*) for each task block. A one-way repeated-measure ANOVA was performed to examine differences in performance distraction index under task type. No significant differences were found (*F*(2,19) = 1.85, *p* = 0.17, MS = 0.039, $\eta_{p}^{2}=0.089$). Overall, these findings suggest both distraction indices appear to be consistent within participants across tasks.

### Exploratory Analysis

**FIGURE 6 |:**
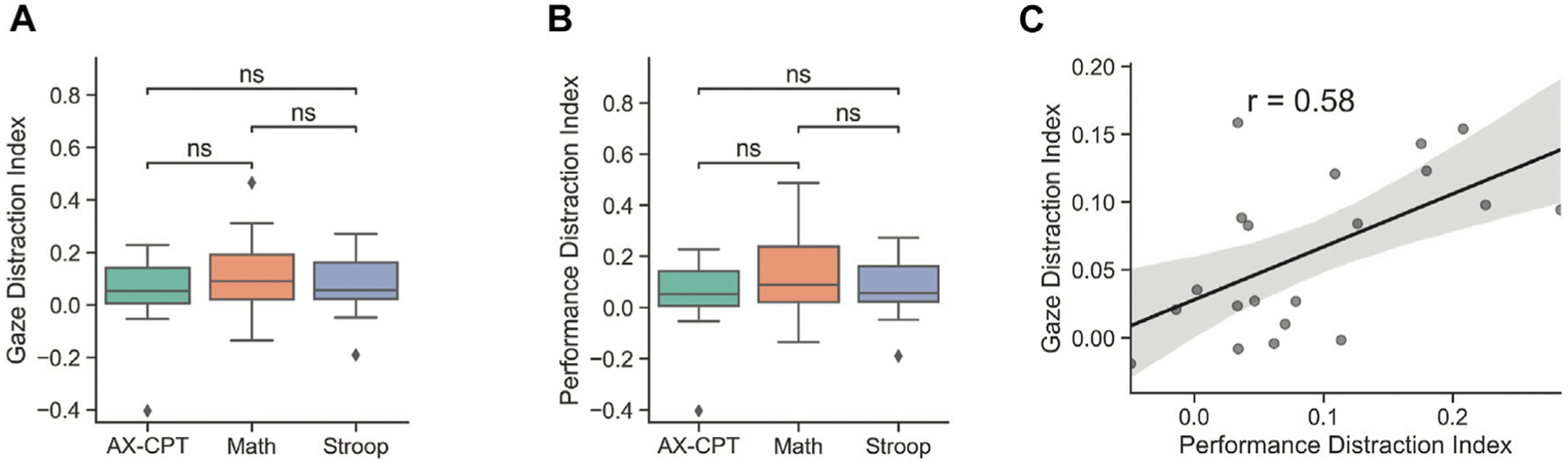
Eye gaze by Task Performance. **(A)** Gaze distraction index by task. **(B)** Performance distraction index by task. **(C)** Correlation between gaze and performance distraction indices (r(19) = 0.58, p < 0.05).

Next, we explored the relationship between gaze and distraction performance indices at the subject level (*n* = 20). Results indicate that there was a significant positive correlation between mean gaze and performance indices (*r*(19) = 0.58, *p* < 0.05). To analyze the relationship between gaze and task performance, we investigated how response rate predicted gaze distraction index using a linear mixed model. Gaze distraction index was set as the dependent variable and response rate as the independent predictor. Subject, task type, and distractor type were included as random effects. There was a significant main effect of response rate (β= −0.15, SE = 0.052, t(113) = 4.89, *p* < 0.005). This shows that that lower response rates were related to a higher gaze distraction index (Figure 6 Distractors). This analysis suggests response rates and higher rates of eye gaze away from the whiteboard reflect a common cause of distraction.

## DISCUSSION

Similar to Mangalmurti et al. (2020) and Lev et al. (2020) we found that eye-tracking during attention-demanding tasks, whether in a VR environment or traditional CPT demonstrate that ADHD is associated with slow disengagement from distractors, or non-target, information. Our project also adds to the literature that suggests eye-tracking, particularly in the context of VR, has the potential to further our understanding of attentional impairments in response to distractors and serve as a biomarker to assess treatment effects. This study is novel in that we are able to extend previous findings (Mangalmurti et al., 2020) to demonstrate over a time course, and replicated across a variety of attention-demanding tasks, that when children are distracted, and disengaged from a target task (i.e., work on the whiteboard) they not only look at the distractor, but their attention is captured by a multitude of other objects and actions in the room. Eye-tracking in a virtual environment enables us to detect patterns of distraction, the type(s) of object or event that are distracting, the duration each target is distracting, along with other rich information to help us better understand the nature of distraction for children with ADHD. Our findings that once children are distracted by a distracting event and that they continue to be dis-engaged for a significant time period while their attention is captured by other stimuli, beyond the initial distractor, suggests that teachers may need to rethink strategies for re-engaging the child with ADHD beyond the obvious distractors.

The combined technology of eye-tracking and VR environments have the potential to help us detect on an individual level which type of objects and activities might be most distracting for a child and the sequence of the eye movements to the various distractors. This will enable more personalized interventions in the future. Our study found that participants were frequently most distracted by the child avatars that were close in proximity to their VR seat but were also highly distracted by events occurring outside the VR class window. Future reports from our laboratory will present those findings in greater detail.

We selected three tasks to test our hypotheses, with the CPT, being the one most studied in the VR-ADHD literature (Parsons et al., 2007; Adams et al., 2009; Bioulac et al., 2020), but added another traditional task used in ADHD, the Stroop task (Barkley et al., 1992), as well as a task requiring the completion of a series of math fluency problems. Where CPTs have been designed by researchers to directly assess sustained attention, the Stroop task requires the participants to inhibit conflicting stimuli, a function more difficult for children with ADHD (Scheres et al., 2004). Importantly, we used a version of the Stroop that presented auditory and visual stimuli, that required good attention to the auditory information, which can be particularly challenging for children with ADHD. In addition to potentially boosting the ecological validity of the classroom experience by requiring speech perception, even minimally, auditory-sustained attention could impact reactions to the audio components incorporated into the virtual classroom distractors. Our Math task was included to simulate an activity often associated with a classroom setting. Often students with ADHD often have difficulty with math (Loe and Feldman, 2007), a subject that could serve as an academic outcome measure for the evaluation of treatment programs. These difficulties can include performing fewer problems and making more errors as well as lower standardized test scores. Our inclusion of a math task in addition to standard attention tasks (i.e., CPT and Stroop), is innovative in that it simulates a traditional classroom task. We recommend that future VR projects use tasks that are representative of what a child would normally experience in a standard classroom to enhance the ecological validity of the VR scenario for studies attempting to perform interventions within the VR environment.

Collectively we aimed to simulate real-world activity in the classroom and found that the distractors decreased attention across all three tasks. Overall, the eye-tracking and performance measures were strikingly similar across the three tasks, suggesting that the VR classroom with distractors is a robust system for detecting real-time distraction, regardless of the task. Eye movement measures have been shown in past studies to successfully task-relevant behavior in children with ADHD. However, few studies have investigated the potential of VR applications with eye gaze tracking for children with ADHD. We chose to use response rate as a performance measure of attention on task and task completion. In addition to being the most feasible score to compare across tasks, task completion is one of the most concerning aspects of performance for parents and teachers of children with ADHD, during classroom time, homework completion, and general functioning. In ADHD, the concern is often less of whether a child is able to perform the task, but more with whether they will actually do it and do so in a timely manner. For example, the “Permanent Product Measure of Performance,” is a math test used in pharmacological trials with ADHD to assess the effectiveness of medication by assessing the number of math problems completed (Wigal and Wigal, 2006). Thus, the sensitivity of the rate of performance is a measure that is likely to have good generalizability. Future studies should assess how well measures of rate in the VR classroom relate to measures of task completion in the real-world setting. Furthermore, there may be individual differences in how interest in the tasks affected engagement, for example we saw some children very much enjoyed the math tasks, while others did not. While we found similar findings in our results across tasks, we recommend future studies evaluate if the interest in the task may impact the results.

There were several limitations in this proof of principle project, including a small sample size and thus, future studies should include a greater number of participants. Participants in our study were not currently taking medication for ADHD, which could suggest that the children had less severe ADHD symptoms. However, California has one of the lowest rates of prescribing medication for ADHD in the country (Visser et al., 2013); the fact that the subjects were not medicated may also reflect that they were California residents. Future studies could assess the relationship between severity of ADHD symptoms and patterns of eye-tracking in the VR context. This proof of principle project also did not contrast ADHD performance to a healthy comparison group, without ADHD. The VR classroom has repeatedly shown significantly worse performance in behavior our previous studies (e.g., Parsons et al., 2007; Adams et al., 2009) and others (e.g., Mangalmurti et al., 2020), suggesting that that these effects would likely discriminate between groups. We consider this project to be the first in the series of projects and recommend future studies conduct testing between ADHD and typically developing children to assess differences in eye-tracking performance within the VR classroom.

The formal validation of using eye-tracking and VR in assessing distractibility will require larger scale efforts including test-retest reliability, convergent validity amongst samples of children in a broader age span and with degrees of impairing distractibility to confirm its utility. There is significant potential to use VR-eye tracking measures as eye gaze measurement technology is being integrated into many of the newer VR headsets at fairly affordable costs. With these advances eye-tracking in the VR context will have increased potential to assess treatment effects on distractibility and likely across a variety of settings. It might even be possible for some of the testing to occur in the child’s home environment, in the future.

Future projects will assess the potential for the delivery of treatments using VR-based technology, including for distractibility and other symptoms associated with ADHD. The literature suggests that training within the VR context for ADHD has potential in a small meta-analysis (Romero-Ayuso et al., 2021). The VR technology also permits a wider range of scenes that could be developed for use with adolescents and adults (e.g., office) in a variety of settings. Ultimately, however, the greatest potential for intervention within the VR context is to demonstrate that these intervention effects will generalize to the day-to-day environment and be sustained over time.

## Supplementary Material

Supplementary_Material_Measuring Attentional Distraction in Children With ADHD Using Virtual Reality Technology With Eye-Tracking

## Figures and Tables

**TABLE 1 |:** Demographic and clinical characteristics of participants.

		Number
**Sex**		
Female		2
Male		18
**Ethnicity**		
Hispanic		5
Non-Hispanic		15
**Race**		
American Indian or Alaskan Native		0
Asian		2
Black or African American		2
Native Hawaiian or Pacific Islander		0
White		10
Other		2
Unknown		0
Multiracial		4
	Mean	SD
Age	10	1.65
**Conners Parent Rating Scale**^a^		
Inattention T-Score	76.375	8.823
Hyperactive/Impulsive T-Score	76.375	13.09
**WIAT**		
Word Read SS	123.72	24.8
Numerical Operation SS	97.77	14.72
**WASI II**		
Vocab T-Score	51.105	9.37
Matrix Reasoning T-Score	51.526	10.107
Full Scale IQ-2	102.105	13.908

WIAT, Wechsler Individual Achievement Test; WASI II, the Weschler Abbreviated Scale of Intelligence.

a T-scores 65 or > are considered clinically significant on the Conners.

**TABLE 2 |:** Distractors.

Distractor stimuli	Distraction	Duration (s)	Location in the virtual classroom
Student avatar 1			
	Sneezing	6.7	Front of classroom left
	Tapping	8	
Student avatar 2			Front of the classroom right
	Humming	11.3	
	Dropping pencil	8	
Student avatar 6			Front-Right
	Whispering	5.8	
	Tapping	8.33	
Student avatar 8			Directly left
	Drops Pencil	8	
Student avatar 9			Directly right
	Whispering	5.8	
	Tapping		
School Bus			Left
	Passing up and down the street	11.1	
Phone			Right/Front-Right
	Ringing	7.9	
Pedestrian			Left
	Walking by window	20	
Car			Left
	Passing up and down the street	5.2	

## Data Availability

The raw data supporting the conclusions of this article will be made available by the authors, without undue reservation.
