# Supplementary material for: Measuring Attentional Distraction in Children With ADHD Using Virtual Reality Technology With Eye-Tracking
Source: Front Virtual Real. Author manuscript; Available in PMC 2022 May 19. (PMC9119405; doi:10.3389/frvir.2022.855895)
Supplement: Supplementary_Material_Measuring Attentional Distraction in Children With ADHD Using Virtual Reality Technology With Eye-Tracking [file NIHMS1805148-supplement-Supplementary_Material_Measuring_Attentional_Distraction_in_Children_With_ADHD_Using_Virtual_Reality_Technology_With_Eye-Tracking.pdf]

Supplementary Table 1. Full ANOVA results.

Eye Gaze

Task	Source	SS	DF	MS	F	p-unc	np2	eps
Stroop	bin_id	0.054	2	0.027	8.487	0.001	0.309	0.857
	Error	0.12	38	0.003				
Math	bin_id	0.077	2	0.038	12.856	0	0.404	0.873
	Error	0.114	38	0.003				
AX-CPT	bin_id	0.023	2	0.012	6.788	0.003	0.263	0.72
	Error	0.065	38	0.002				

Task Performance

Source	SS	Ddof1	Ddof2	MS	F	p-unc	p-GG-corr	np2	eps
Task_type	0.649	2	38	0.325	68.313	0	0	0.782	0.846
Distractor_state	0.014	1	19	0.014	19.753	0	0	0.51	1
Task_type * distractor_state	0.003	2	38	0.001	3.3	0.048	0.053	0.148	0.906

Gaze Distraction Index

Source	SS	DF	MS	F	p-unc	np2	eps
Task_type	0.012	2	0.006	2.149	0.131	0.102	1
Error	0.107	38	0.003				

Performance Distraction Index

Source	SS	DF	MS	F	p-unc	np2	eps
Task_type	0.077	2	0.039	1.85	0.171	0.089	0.877
Error	0.795	38	0.021				
